# Nanoengineered t-ZrO_2_ coatings for superior corrosion resistance on steel surfaces

**DOI:** 10.1038/s41598-025-30044-y

**Published:** 2025-12-01

**Authors:** H. Mohamed Kasim Sheit, K. S. Mohan, N. Geetha, R. Lavanya, Karthik Kannan, S. Esakki Muthu, Manikandan Ayyar, Prabhu Paramasivam, Saravanan Rajendran, M. Santhamoorthy, S. Santhoshkumar, Ankush Mehta

**Affiliations:** 1 PG and Research, Department of Chemistry, Jamal Mohamed College (Autonomous), Affiliated to Bharathidasan University, Tiruchirappalli, Tamil Nadu 620 020 India; 2 Department of Physics, Nandha Engineering College, Erode, Tamil Nadu 638 052 India; 3 Department of Chemistry, Paavai Engineering College (Autonomous), Namakkal, Tamil Nadu 637 018 India; 4 Department of Chemistry, Sri Sairam Engineering College (Autonomous), West Tambaram, Chennai, Tamil Nadu 600 044 India; 5 Advanced Institute of Manufacturing With High-Tech Innovations and Department of Mechanical Engineering, National Chung Cheng University, Chia-Yi, 621301 Taiwan; 6 Centre for Materials Science, Karpagam Academy of Higher Education, Coimbatore, Tamil Nadu 641021 India; 7 Department of Physics, Karpagam Academy of Higher Education, Coimbatore, Tamil Nadu 641021 India; 8 Department of Chemistry, Karpagam Academy of Higher Education, Coimbatore, Tamil Nadu 641021 India; 9 Centre for Material Chemistry, Karpagam Academy of Higher Education, Coimbatore, Tamil Nadu 641021 India; 10 Centre for Research Impact & Outcome, Chitkara University Institute of Engineering and Technology, Chitkara University, Rajpura, Punjab 140401 India; 11 Instituto de Alta Investigación, Universidad de Tarapacá, 1000000 Arica, Chile; 12 School of Chemical Engineering, Yeungnam University, Gyeongsan, 38541 Republic of Korea; 13 Department of Biochemistry, Saveetha Medical College and Hospital, Saveetha Institute of Medical and Technical Sciences, Chennai, Tamil Nadu India; 14 Marwadi University Research Center, Department of Mechanical Engineering, Faculty of Engineering & Technology, Marwadi University, Rajkot, Gujarat 360003 India; 15 Department of Mechanical Engineering, Mattu University, 318, Mettu, Ethiopia

**Keywords:** Green synthesis, *Acacia nilotica*, t-ZrO_2_ NPs, Anticorrosion study, Chemistry, Materials science, Nanoscience and technology

## Abstract

Corrosion of steel has several catastrophic consequences in various sectors. The inorganic nanoparticle-based anticorrosive coating on steel drew important attention to its large surface-to-volume ratio. The primary aim of this study is to synthesize and characterize t-ZrO_2_ nanoparticles at an optimized annealing temperature and evaluate their structural, morphological, and optical properties. Additionally, the study investigates their effectiveness as a corrosion inhibitor for carbon steel in 1 M H_2_SO_4_. The study explores the cheap, facile, green synthesis of t-ZrO_2_ nanoparticles (NPs) through bark extract from the gum arabic plant (*Acacia nilotica*) for anticorrosive coatings on carbon steel. X-ray diffraction (XRD) analysis confirms the tetragonal phase structure and the crystallite size, calculated using Scherrer’s formula, is found to be 8.1 nm. Fourier-transform infrared (FT-IR) spectroscopy reveals the presence of Zr-O bonding along with organic residues from plant extracts, confirming the formation of t-ZrO_2_ NPs. Field emission scanning electron microscopy (FESEM) images confirm a rock stone-like structure, while energy dispersive X-ray (EDX) spectroscopy verifies the presence of Zr and O elements. The study further investigates the corrosion inhibition efficiency of t-ZrO_2_ NPs on carbon steel in 1 M H_2_SO_4_. The atomic force microscopy (AFM) analyses reveal a smoother surface with reduced roughness in the presence of the inhibitor. Electrochemical measurements, including weight loss, potentiodynamic polarization, and electrochemical impedance spectroscopy (EIS), confirm a significant reduction in corrosion rate. The inhibition efficiency reaches 95.2% at 200 ppm of 0.2 M t-ZrO_2_ NPs, with an increased charge transfer resistance (R_ct_) of 14,715 Ω cm^2^ and a reduced double-layer capacitance (C_dl_) of 0.631 × 10⁸ F/cm^2^. These findings demonstrate that t-ZrO_2_ NPs act as an effective corrosion inhibitor for carbon steel in acidic environments.

## Introduction

Nanotechnology, which involves deploying substances at the atomic and molecular scale, has introduced a new age of materials with unique properties that differ significantly from their bulk counterparts. Among these materials, nanoparticles stand out due to their exceptional chemical, mechanical, thermal, magnetic, and electrical characteristics, attributed to their quantum effects with high surface area-to-volume ratio^1,2^. These distinctive features make nanoparticles a subject of intense study and a cornerstone of various technological advancements^3,4^.

The study of metal oxide nanoparticles, particularly those involving the combination of two or more metals, has garnered significant interest^5^. These nanoparticles exhibit a broad spectrum of physical and chemical properties, making them suitable for a variety of uses, from catalysis to electronics and biomedical fields^6^. Zirconium dioxide (ZrO_2_), known as zirconia, is a ceramic material with excellent mechanical properties, chemical stability, and biocompatibility^7^. Its nanoparticle form further enhances these properties, making it a valuable material in numerous high-tech applications, such as oxygen sensors, fuel cells and dental implants^8–10^.

Zirconium, a transition metal similar to titanium, is characterized by its remarkable resistance to corrosion, making it an ideal material for applications in harsh environments^11^. Naturally occurring zirconium can be processed into nanoparticles using various methods. These nanoparticles are noted for their high fracture toughness, tensile strength, and hardness^12,13^. The metal’s natural isotopic forms, particularly the dominant 90Zr, play a crucial role in its industrial applications^14^. The synthesis of zirconium nanoparticles can be achieved through multiple techniques, including the Kroll process, which involves reducing zirconium tetrachloride with magnesium at high temperatures^15^. Other methods, such as sol–gel, hydrothermal, and laser ablation, are also utilized to produce ZrO_2_ NPs in various phases, including cubic and monoclinic forms^16^.

Nanotechnology faces several challenges, including precise control over nanoparticle size, shape, and distribution and ensuring the reproducibility and scalability of synthesis methods^17,18^. The unique properties of nanoparticles can lead to new functionalities, but they also pose potential risks, such as toxicity and environmental impact. Therefore, understanding and controlling these properties are crucial for the safe and effective use of nanomaterials^19^. This research seeks to advance the understanding of zirconium nanoparticles by developing eco-friendly synthesis methods and evaluating their anticorrosive properties on carbon steel in acidic conditions^20^. The use of t-ZrO_2_ NPs in anticorrosion coatings is significant due to their excellent chemical stability, high thermal resistance, and ability to form a dense, protective barrier on metal surfaces. These nanoparticles reduce corrosion rates by preventing direct contact between aggressive electrolytes and the metal substrate. Additionally, their high surface area enhances adhesion properties, ensuring a uniform and defect-free coating. The incorporation of t-ZrO_2_ NPs in protective coatings offers an eco-friendly and efficient approach to mitigating corrosion in industrial applications, extending the lifespan of metal components, and reducing maintenance costs. This study aims to refine the production method to achieve higher synthesis efficiency, better structural and functional properties of the nanoparticles, and reduced environmental impact. In addition, it explores new application areas for zirconium nanoparticles, with a special emphasis on high-performance anticorrosive uses^21,22^.

## Experimental methods

### Chemicals used

Zirconium Dichloride Oxide (ZrOCl_2_⋅8H_2_O) analytical grade purchased from Sigma–Aldrich (99% purity by weight) is used without further purification.

#### Plant material collection

The *Acacia nilotica* bark extract powder used in this study was obtained from Dr. A. Aslam (Fellow of the Indian Association for Angiosperm Taxonomy), Associate Professor of Botany, Jamal Mohamed College (Autonomous), Tiruchirappalli, Tamil Nadu, India.

### Preparation of t-ZrO_2_ NPs by means of ***Acacia nilotica*** bark extract

**Fig. 1:**
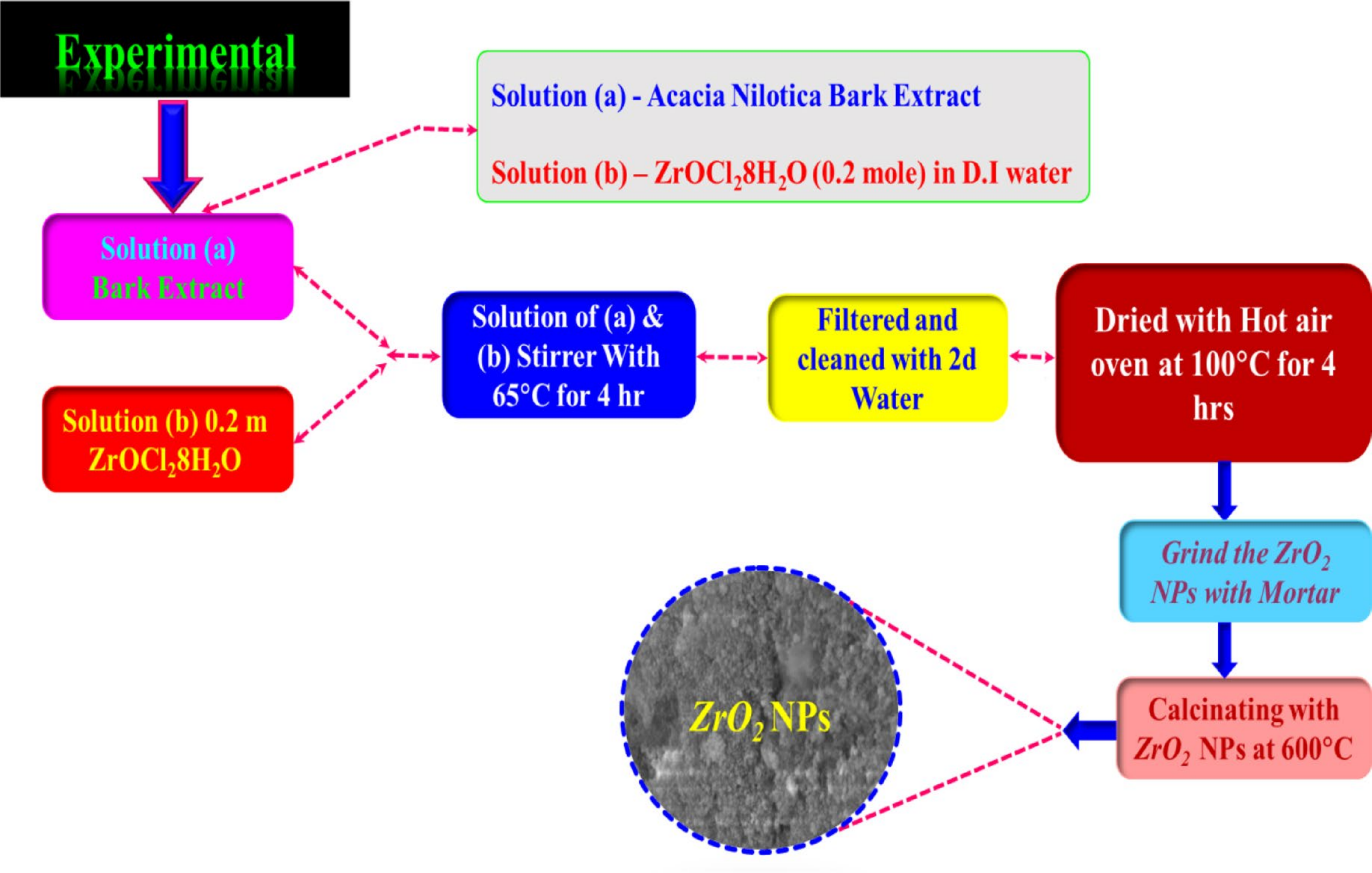
A structured method for the process of chemical reduction root of a t-ZrO_2_ NPs for 0.2 mol concentrations.

To prepare the aqueous bark extract, 100 mL of double-distilled water and 10 g of finely chopped *Acacia nilotica* bark were heated at 60 °C for 30 min to extract the bioactive compounds, followed by filtration through Whatman No. 1 filter paper to obtain the aqueous extract used for the nanoparticle synthesis. A 0.2 M solution of zirconium dichloride oxide (ZrOCl_2_·8H_2_O) was prepared and gradually added to the *Acacia nilotica* bark extract under constant stirring to facilitate the green synthesis of t-ZrO_2_ nanoparticles. After four hours of stirring at 65 °C, a white precipitate formed from this combination. To avoid agglomeration, the precipitate was separated using filtration, cleaned with distilled water, and dried for four hours at 100 °C. At 600 °C, the dried powder was finally calcined. Figure 1 displays the schematic representations. In this study, a 0.2 M precursor concentration of ZrOCl_2_·8H_2_O was selected based on preliminary synthesis trials and previous reports indicating that this concentration yields zirconia nanoparticles with optimal crystallinity and phase stability for t-ZrO_2_ systems^23–29^.

### Specimen preparation for carbon steel

Carbon steel specimens were obtained from a single sheet with the following composition: 2% carbon, 0.06% phosphorus, 0.026% sulfur, and 0.4% manganese, with the remainder being iron. Each specimen, measuring 1.0 × 4.0 × 0.2 cm, was polished to a mirror-like finish and cleaned with trichloroethylene before use in surface analysis and weight-loss corrosion testing. For potentiodynamic polarization experiments, a carbon steel specimen was mounted in a Teflon holder, exposing a 1 cm^2^ cross-sectional area as the working electrode, while a platinum electrode was used as the counter electrode. Both electrodes were degreased with trichloroethylene and polished to a mirror finish. Analytical-grade sulfuric acid was diluted using double-distilled water to create a 1 M H_2_SO_4_ solution.

### Formulating stock solutions

All solutions are made using deionized water and premium H_2_SO_4_ is diluted to the required concentration. The inhibitor is dissolved directly in this acidic medium, and the solution is then adjusted to reach the target concentration.

### Characterization techniques

Before and after being submerged in 1 M H_2_SO_4_ for three hours, three polished carbon steel (CS) specimens were weighed with different extract quantities to determine weight loss.

1 $${\text{Inhibition Efficiency}}\;IE(\% ) = \left( {\frac{{W_{o} - W_{1} }}{{W_{o} }}} \right) \times 100\%$$

Weight loss with the inhibitor is represented by W1, and weight loss without the inhibitor is represented by Wo. Using the formula, the corrosion rate is calculated in mmd,

2 $${\text{Corrosion Rate }}\left( {{\text{mdd}}} \right) = \frac{53.5 \times W}{{a \times t}}$$

where W stands for weight loss (g), *a* for specimen area (cm^2^) , and t for exposure time (h).

**Fig. 2:**
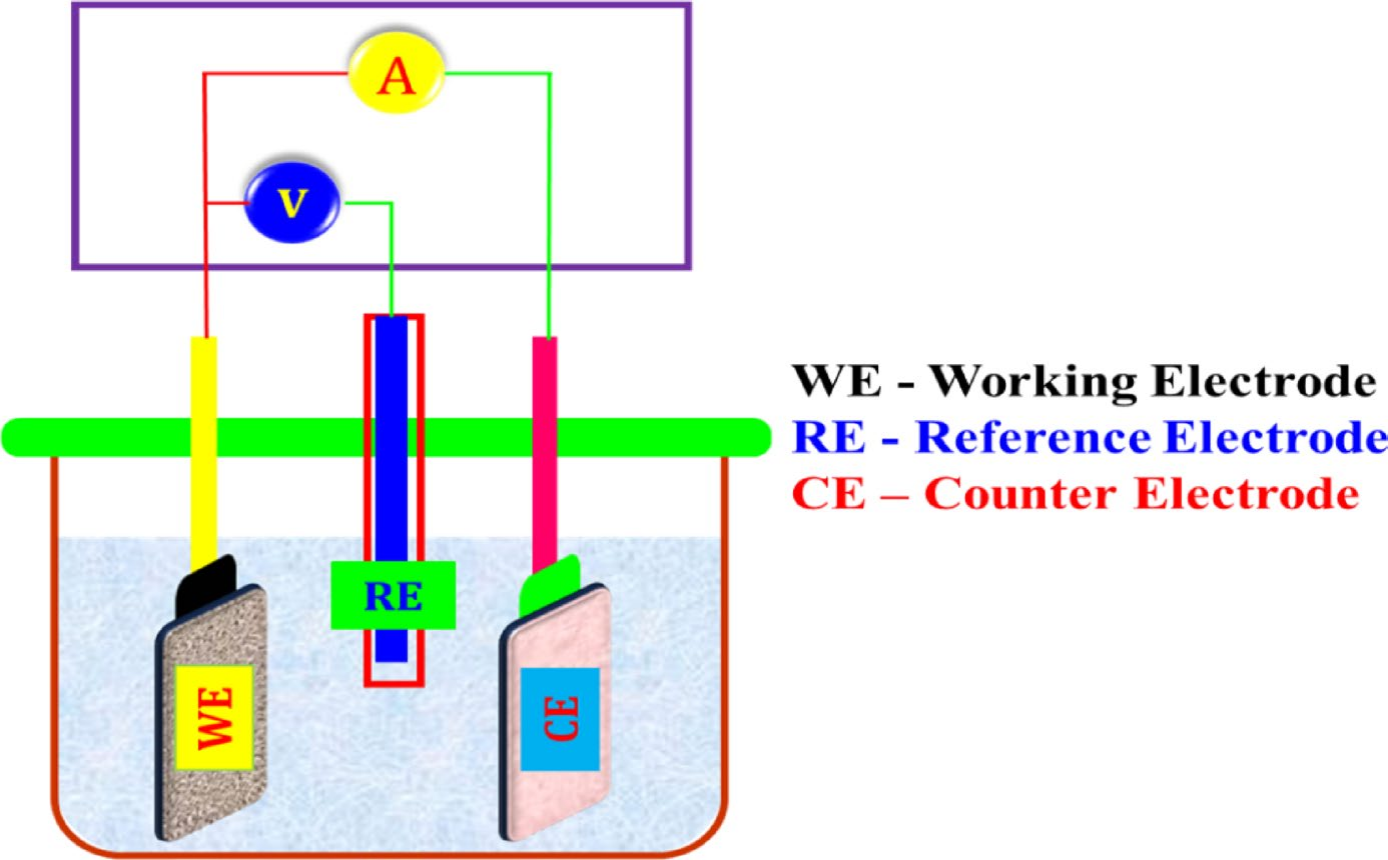
Schematic diagram of a three-electrode cell assembly.

Electrochemical Impedance Spectroscopy (EIS) measurements were carried out in a frequency range spanning from 1 Hz to 100 kHz (10^5^ Hz) with an applied AC amplitude of 0.005 V, a starting voltage of 0 V, and a 2-s quiet period before measurement, using a CHI 660 A electrochemical workstation. The experiment used a three-electrode cell setup, as shown in Fig. 2. The saturated calomel electrode (SCE) act as a reference electrode, and platinum electrode was used as the counter electrode. Carbon-based steel was used as an electrode. LPR values were derived from the polarization investigation, and the polarization analysis under consideration was able to compute corrosion parameters suggestive of rust current (I_corr_), rust potential (E_corr_), and Tafel slopes (anode = b_a_ and cathode = b_c_). Using a polarization analyzer, the AC impedance spectra were evaluated.

In order to describe a system’s resistance to AC flow, Measurements of impedance are taken at every frequency within the specified interval. The following formula is employed to determine the value of the double-layer capacitance.

3 $$C_{dl} = \frac{1}{{(2\pi \times f_{\max } \times R_{ct} )}}$$

where, C_dl_ is the double-layer capacitance (F/cm^2^), Rt (more appropriately denoted as R_ct_) is the charge transfer resistance (Ω cm^2^) and fmax is the frequency at which the imaginary component of impedance (Z″) reaches a maximum. The real (Z′) part and imaginary (Z″) parts, given in ohms, were taken into consideration when analyzing the cell’s resistance at different frequencies. The values of charge transfer resistance (R_ct_) and double-layer capacitance (C_dl_) were calculated using the Nyquist plot. Using Bode graphs, the logarithmic (log) impedance value (z/ohm) was determined.

The inhibition efficiency EIS studies are calculated using following formulas

4 $${\text{IE}}(\% ) = \frac{{{\text{i}}_{{{\text{Corr}}}} - {\text{i}}_{{{\text{inh}}}} }}{{{\text{i}}_{{{\text{Corr}}}} }} \times 100$$

where, i_corr_ -corrosion current density without inhibitor and i_inh_ -corrosion current density with inhibitor

5 $${\text{IE}}(\% ) = \frac{{{\text{R}}_{{{\text{ct}}{\text{.inh}}}} - {\text{R}}_{{{\text{ct}}}} }}{{{\text{R}}_{{{\text{ct}}{\text{.inh}}}} }} \times 100$$

where, R_ct,inh_—charge transfer resistance with inhibitor and R_ct_—charge transfer resistance without inhibitor.

FTIR spectroscopy was conducted Utilizing a Perkin Elmer spectrophotometer within the 400–4000 cm^−1^ range. After removing the protective coating, the CS samples were exposed to the plant leaf extracts and let soak in 1 M H_2_SO_4_ at RT for three hours. Scanning electron microscopy (SEM) studies were performed before and after corrosion to evaluate how the inhibitor affects the CS surface. The surface topography of the CS was analyzed using SEM both with and without the inhibitor, with images acquired using a JEOL JSM 6390. After corrosion, the CS samples were dried after being cleaned with double-distilled water. Three hours later, we tested in both inhibitor and blank solutions.

## Results and discussion

### XRD analysis

**Fig. 3:**
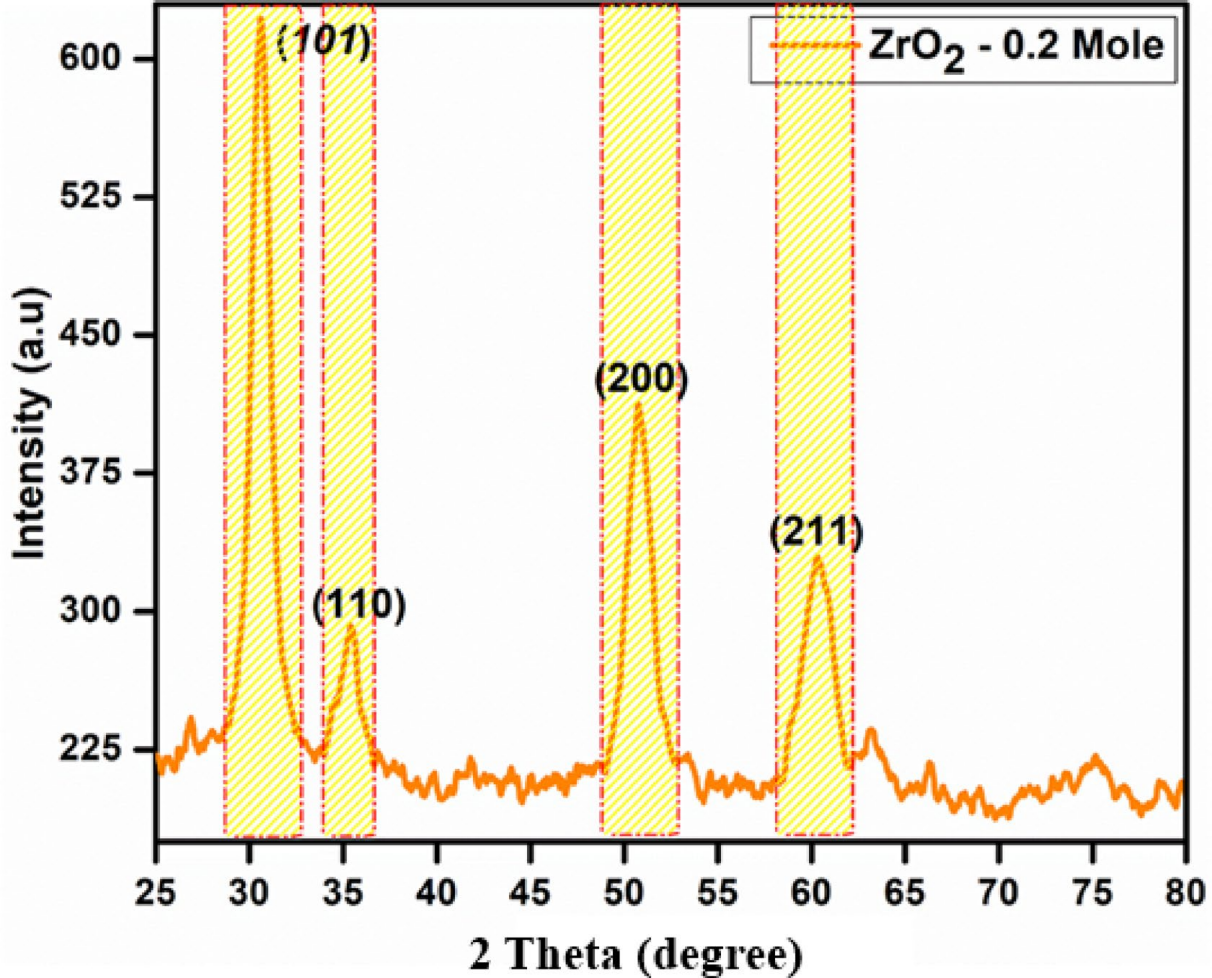
XRD pattern of the t-ZrO_2_ NPs for 0.2 mol concentrations.

Figure 3 presents the XRD pattern of the t-ZrO_2_ NPs at 0.2 mol concentration. The diffraction peaks get more intense and the maximum intensity observed in 0.2-mol concentration at an optimized annealing temperature of 600 °C. The XRD comparison is made against standard bulk data (JCPDS card no. 88–1007). However, the nanoscale nature of the synthesized t-ZrO_2_ NPs leads to size- and surface-induced broadening and minor peak shifts. These deviations are accounted for by the calculated crystallite size (8.1 nm), and the presence of strain and stacking faults further confirms the influence of nanoscale effects on structure. The composition of t-ZrO_2_ is established by the diffraction angle of the peaks at 30.52, 35.19, 50.65, and 60.60° and their corresponding (*h k l*) planes (101), (110), (200) and (211) respectively. No phase change was observed in t-ZrO_2_ NPs with 0.2 mol concentration. Changes in the crystallite size and lattice parameter, however, are responsible for a minor deformation in the NPs’ structure^23–25^. At 0.2-mol concentration, a preferred orientation along the (101) plane is seen. As shown in Fig. 3, the prominent peak intensity increases progressively. These findings suggest that NP production in the (101) direction is significantly influenced by mole concentration^26–29^. Nonetheless, peak height in some planes may be influenced by the crystallites’ preferred orientation. The matching peak height will often rise if additional crystallites align themselves in a single direction. Scherrer’s formula, which is provided in Eq. (6), is utilized to calculate the crystallite size (D) of the t-ZrO_2_ NPs. The dislocation density (δ), microstrain (ε), and stacking fault (SF) are attained by using the following Eq. (7–9)^30^.

6 $${\text{Crystallite Size}}\;D = \frac{{\left( {0.89} \right)\lambda }}{\beta cos\theta }$$

7 $${\text{Dislocation Density}}\;\delta = \frac{1}{{D^{2} }}$$

8 $${\text{MicroStrain}}\;\varepsilon = \frac{\beta cos\theta }{4}$$

9 $${\text{Stacking Fault}}\;SF = \left[ {\frac{{2\pi^{2} }}{{45\left( {3\tan \theta } \right)^{\frac{1}{2}} }}} \right]\beta$$

Here, D represents the crystallite size, λ is the wavelength (0.15418 nm) for CuKα, β denotes the full width at half maximum of the diffraction peak, and θ, the diffraction angle in degrees. The XRD pattern of the 0.2 M t-ZrO_2_ sample confirms the formation of a well-crystallized tetragonal (t-ZrO_2_) phase. The diffraction peaks are sharp and well-defined, indicating good crystalline quality and minimal structural defects. The average crystallite size, calculated using the Debye–Scherrer equation, is approximately 8.1 nm**.** The corresponding microstructural parameters—dislocation density (δ = 1.09 × 10^16^), microstrain (ε = 0.0316), and stacking fault (SF = 0.0113)—suggest a low level of lattice imperfections and confirm the high degree of crystallinity of the synthesized nanoparticles. Overall, the XRD analysis provides clear evidence for the successful synthesis of high-quality tetragonal ZrO_2_ nanoparticles at the optimized 0.2 M concentration.

### Functional group analysis

**Fig. 4:**
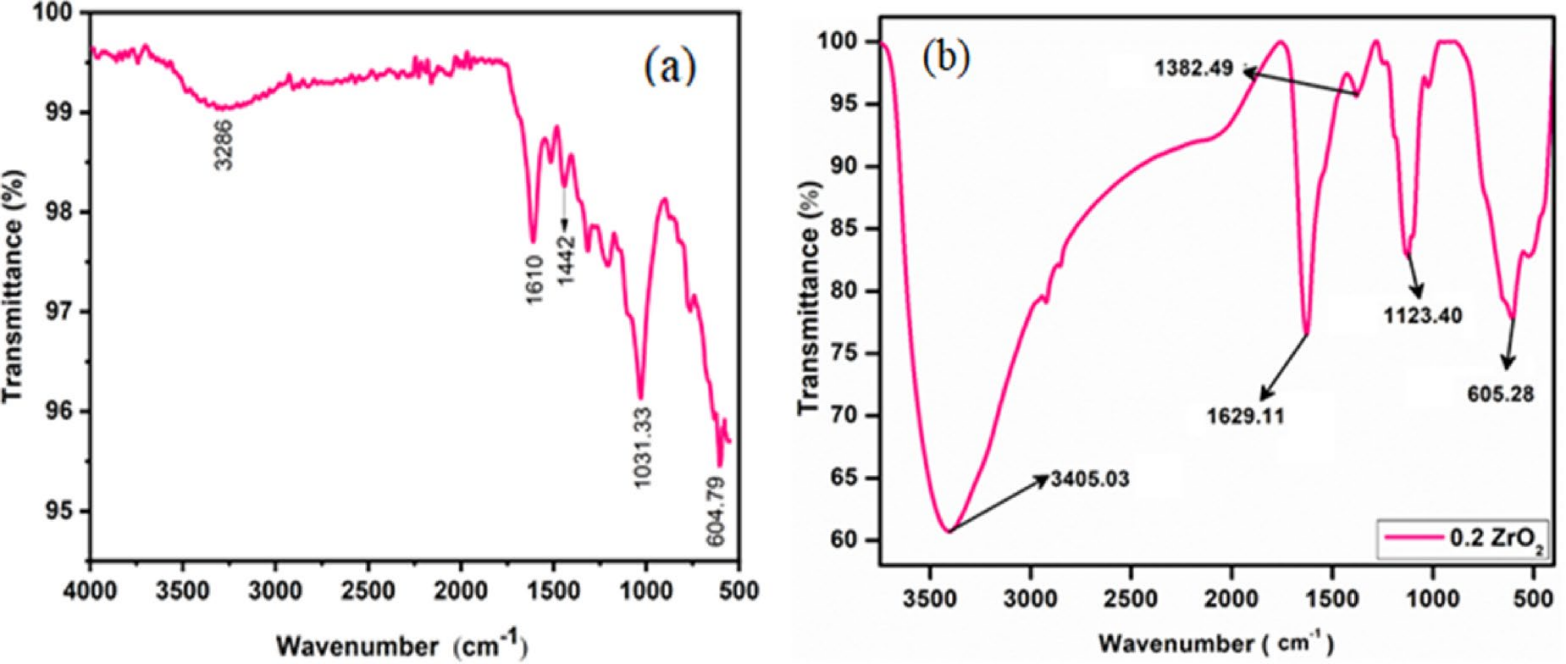
FTIR spectrum of (**a**) Acacia plant bark extract (**b**) t-ZrO_2_ NPs for 0.2 mol concentrations.

The FTIR spectrum of Acacia plant bark extract shows (Fig. 4a ) key absorption bands: 3286 cm^−1^ (O–H/N–H stretching – alcohols, phenols, or amines), 1610 cm^−1^ (C=O or C=C stretching—carbonyl or aromatic/aliphatic double bonds), 1442 cm^−1^ (C–H bending—CH_2_/CH_3_ scissoring), 1031.33 cm^−1^ (C–O–C or C–N stretching—ethers, esters, or amines), and 605 cm^−1^ (aromatic C–H bending or halogen-related C–Cl/C–Br stretch). These indicate the presence of phenolic aromatic structures and possibly halogenated compounds. Figure 4b shows the FT-IR spectrum for the synthesized ZrO_2_ NPs. The band observed at 605.28 cm^−1^ indicates the Zr–O bond stretching vibration in zirconium dioxide. The peak at 1123.40 cm^−1^ suggests C–O stretching vibrations, likely due to residual plant extracts associated with the nanoparticles. The band 1382.49 cm⁻^1^ resembles the symmetric stretching vibration of the C–H bond in -CH_3_ groups, possibly from organic residues of the plant extract. The peak 1629.11 cm⁻^1^ is typically related with C=O stretching vibrations in amides or carbonyl groups, likely originating from organic compounds in the plant extract^31^.

The band at 3405.03 cm⁻^1^ is ascribed to O–H stretching vibrations, signifying the occurrence of hydroxyl groups, which may come from water molecules, alcohols in the plant extract, or surface hydroxyl groups on the nanoparticles. This analysis confirms the formation of ZrO_2_, with the broad peak suggesting the nanoparticles possess a nanostructure^32^.

### UV–Vis spectroscopy analysis

**Fig. 5:**
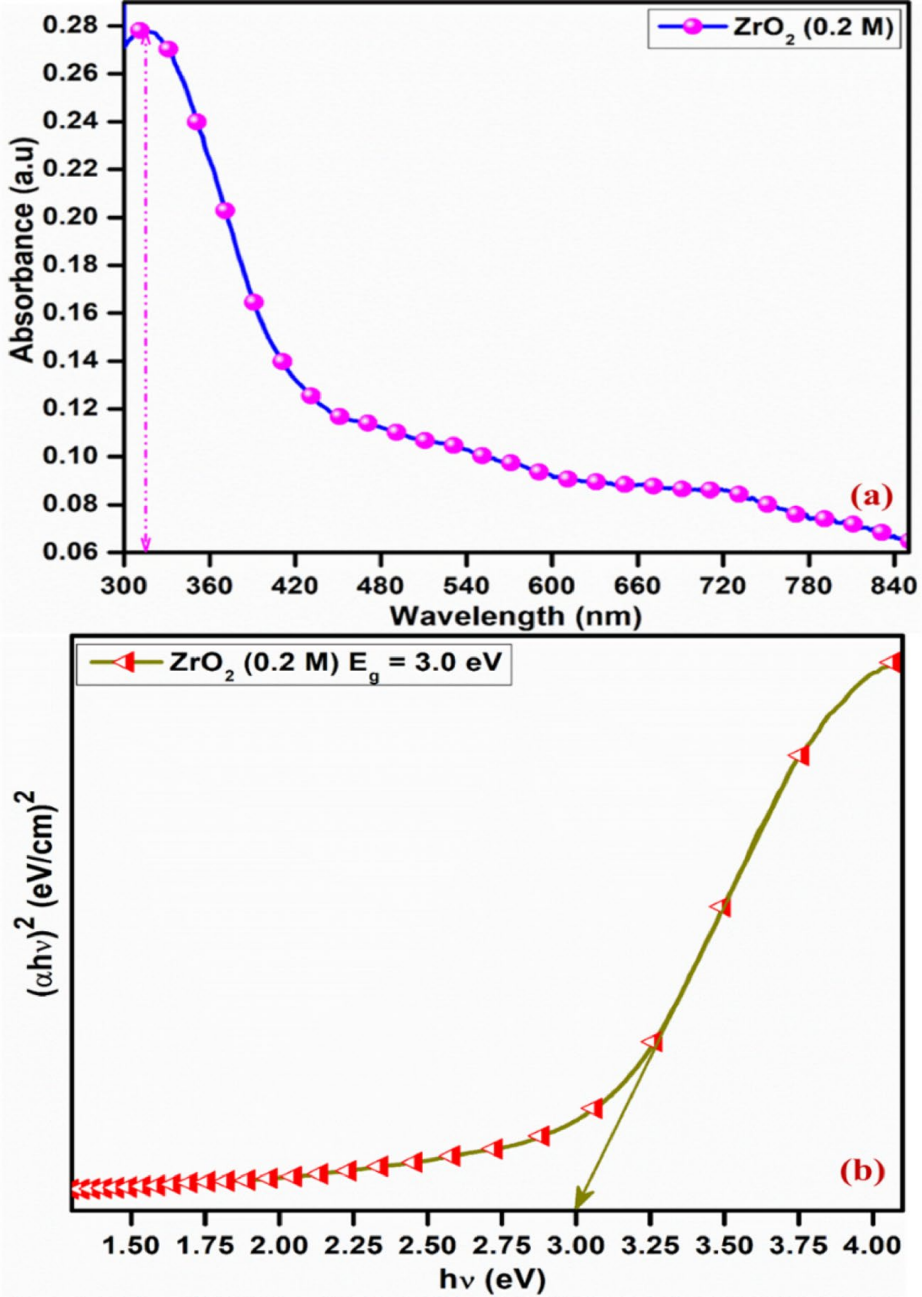
UV–Vis analysis of t-ZrO_2_ NPs for 0.2 mol concentrations (**a**) Absorption (**b**) plots of (αhν)^2^ Vs hν.

Utilizing UV–Vis absorption spectroscopy, the optical characteristics of t-ZrO_2_ NPs were examined. Figure 5a displays the room temperature UV–Vis absorption spectrum of the t-ZrO_2_ NPs. This spectrum corresponds to t-ZrO_2_ NPs at a concentration of 0.2 mol, optimized at an annealing temperature of 600 °C. The UV–Vis absorption band at 317 nm corresponds to the characteristic absorption of t-ZrO_2_ nanograins, influenced by quantum size effects, in contrast to the bulk exciton absorption. This redshift is attributed to specific experimental conditions and defect states introduced during green synthesis^33^. Perhaps as a result of the particular experimental synthesis conditions, the absorption in the visible range suggests that the generated t-ZrO_2_ NPs have some defect energy levels^34^. The Tauc plot determines prepared structures’ optical band gap Eg (Fig. 5b).

According to the following connection, the absorption coefficient in this case depends on the photon energy and band gap (10):

10 $${\text{Band gap}}\;\left( {\alpha h\nu } \right) = A(h\nu - E)^{n}$$

where A is the proportionality constant, $\nu$ is the vibration frequency, Eg, the band gap, h is the Planck constant, and α is the absorption coefficient. When n = 2, it specifies directly allowed transitions and intercepts on the E-axis, giving the values of E_g_. Because of the size effect, the intercept of the curve’s extrapolated linear component has yielded an energy band gap Eg of roughly 3 eV^35^. The green synthesis method minimizes the band gap value of t-ZrO_2_ NPs.

### Surface morphology

#### FESEM analysis of t-ZrO_2_ NPs

**Fig. 6:**
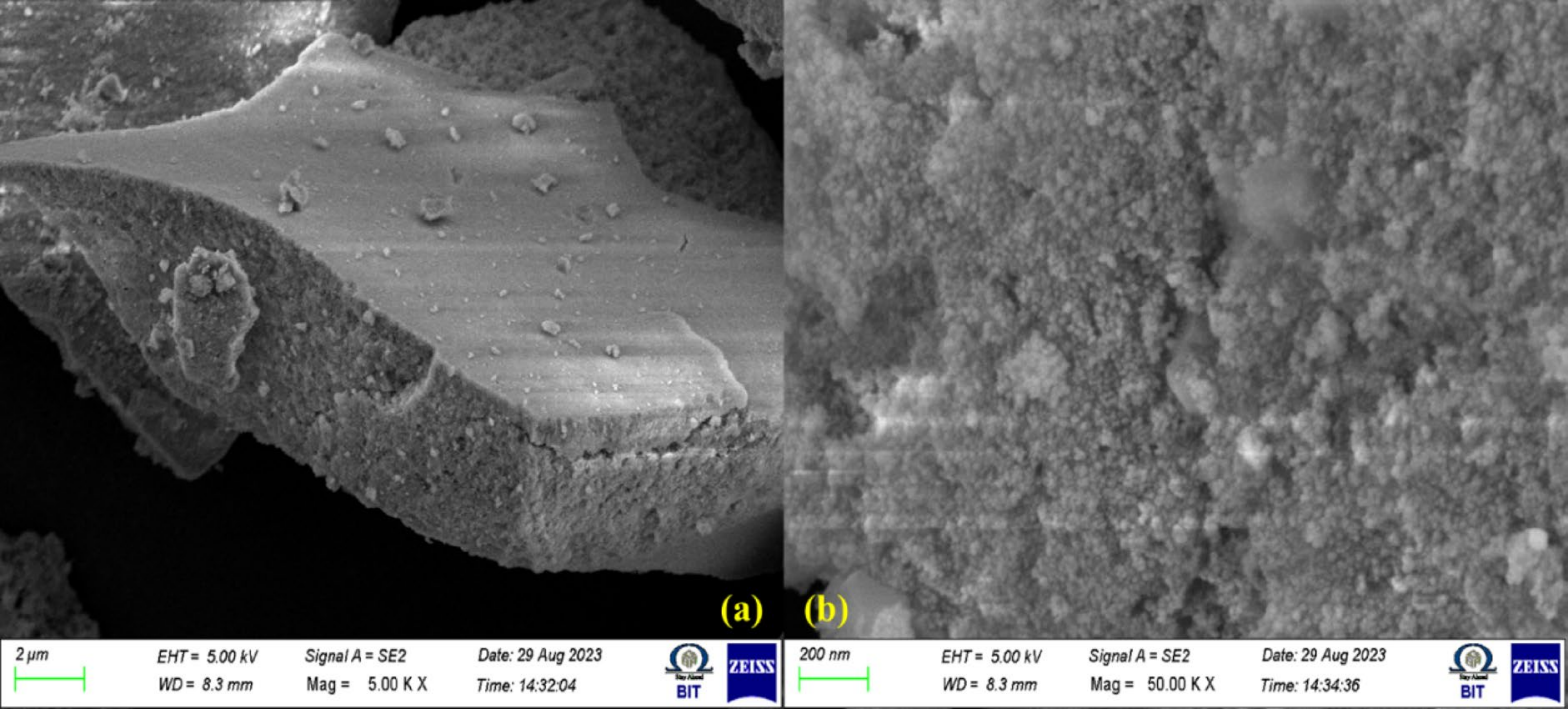
(**a**,**b**) FESEM images of t-ZrO_2_ NPs for 0.2 mol concentrations.

FESEM micrographic images of t-ZrO_2_ nanoparticles for 0.2-mol concentration is shown in Fig. 6a,b, displays the t-ZrO_2_ grains that are micrometer and nanometer in size. The surface morphology exhibits continuous variation in mole concentration on the t-ZrO_2_ nanoparticle surface. FESEM images of t-ZrO_2_ nanoparticles reveal no cracks observed, even in the large area scanning. For the t-ZrO_2_ NPs, the randomly oriented rock stone-like structure is detected. Due to the Zr atoms oxidation, the mole concentration favors quick and defects-free growth crystallites^36–38^. The surface morphology of Zirconium Oxide nanoparticles is studied, and their large surface area makes an increased quantity of atoms on the crystal compound’s surface compared to the atoms inside the crystal. The bulk of t-ZrO_2_ particles is formed by the interaction of these atoms with other contiguous surface atoms or by the absorption of other species. These atoms on the surface of the zirconium oxide nanoparticles have more dandling bonds and free valance bonds. The annealing temperature support to minimize the crystalline size values follows the XRD results^39^.

#### SEM analysis of carbon steel with inhibitor

**Fig. 7:**
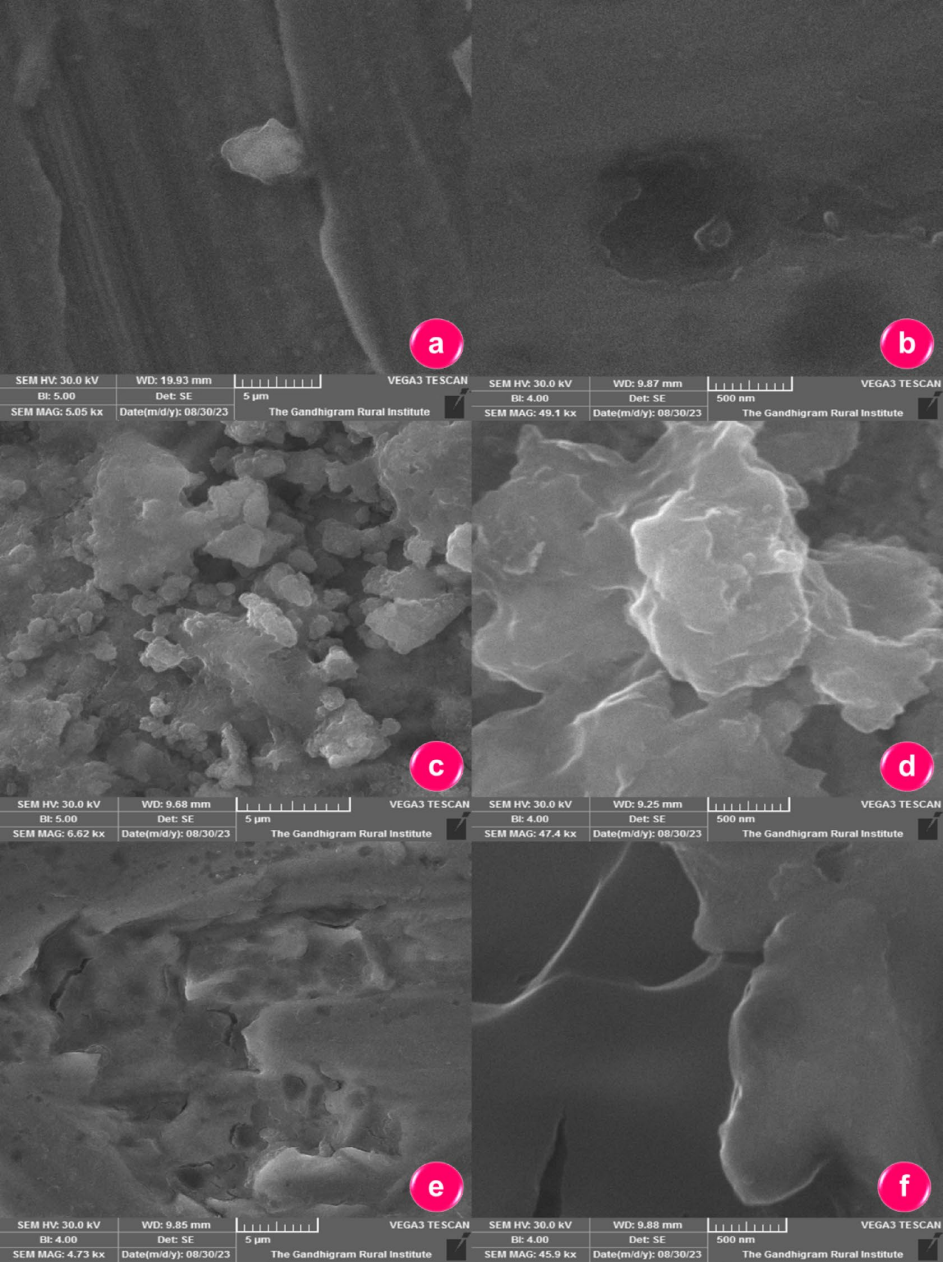
SEM micrographs of (**a**,**b**) polished carbon steel, (**c**,**d**) carbon steel exposed to 1 M H_2_SO_4_, and (**e**,**f**) carbon steel exposed to 1 M H_2_SO_4_ containing 200 ppm of 0.2 M t-ZrO_2_ nanoparticles.

Scanning Electron Microscopy (SEM) provides detailed images of the carbon steel, offering insight into the surface morphology. The study examined the SEM morphology of carbon steel in 1 M H_2_SO_4_ solution, both with and without an inhibitor system. Figure 7a,b represent the polished carbon steel surface, which appears smooth and free from any corrosion features. No pits or deposits are visible, confirming the unexposed, pristine condition of the metal. While Fig. 7c,d corresponds to the blank (uninhibited) carbon steel sample immersed in 1 M H_2_SO_4_. The surface is rough, uneven, and covered with irregular corrosion products. istinct corrosion pits can be observed as dark, circular depressions, indicating localized attack due to acid exposure. Figure 7e,f show the inhibited carbon steel sample exposed to 1 M H_2_SO_4_ + 200 ppm of 0.2 M t-ZrO_2_ nanoparticles. The surface is comparatively smoother with fewer visible defects or pits, suggesting that the inhibitor effectively suppressed corrosion and promoted the formation of a protective film on the steel surface^21^. Hence, the morphology and size of t-ZrO_2_ NPs significantly influence their anticorrosion properties by affecting surface coverage, adhesion, and barrier effectiveness. The observed higher surface area ensures uniform dispersion and reduces coating porosity, which minimizes the penetration of corrosive agents. Additionally, optimized nanoparticle size improves charge transfer resistance, reducing the corrosion rate and enhancing overall coating performance.

### EDX analysis

**Fig. 8:**
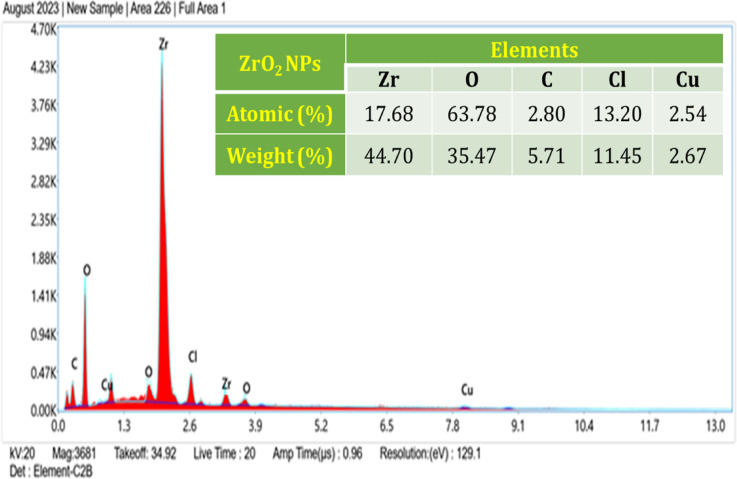
EDX spectrum of t-ZrO_2_ NPs for 0.2 mol concentration.

The elements in the produced samples were examined using energy dispersive spectroscopy. Figure 8 displays the EDS spectrum of the prepared sample. From the figure it can be understood that the sample contains only Zr, O NPs with 0.2 mol concentration. The atomic % of Zr and O are 17.68 and 63.78 t-ZrO_2_ with 0.2-mol concentration. The presence of t-ZrO_2_ NPs in the sample is confirmed by the strong peaks that correspond to Zr and O. The added peaks in EDS spectra are associated with the annealing temperature of 500 °C C, Cl, and Cu used for sample preparation. The annealing temperature causes the Zr content to decrease as the oxygen level increases. From the EDX results, the t-ZrO_2_ NPs specify the strong impact of the Zr lattice at mole concentration. This result discloses a suitable chemical composition with stoichiometry of chemical elements was achieved for the zirconium oxide nanoparticle^17^.

### Atomic force microscopic studies

**Fig. 9:**
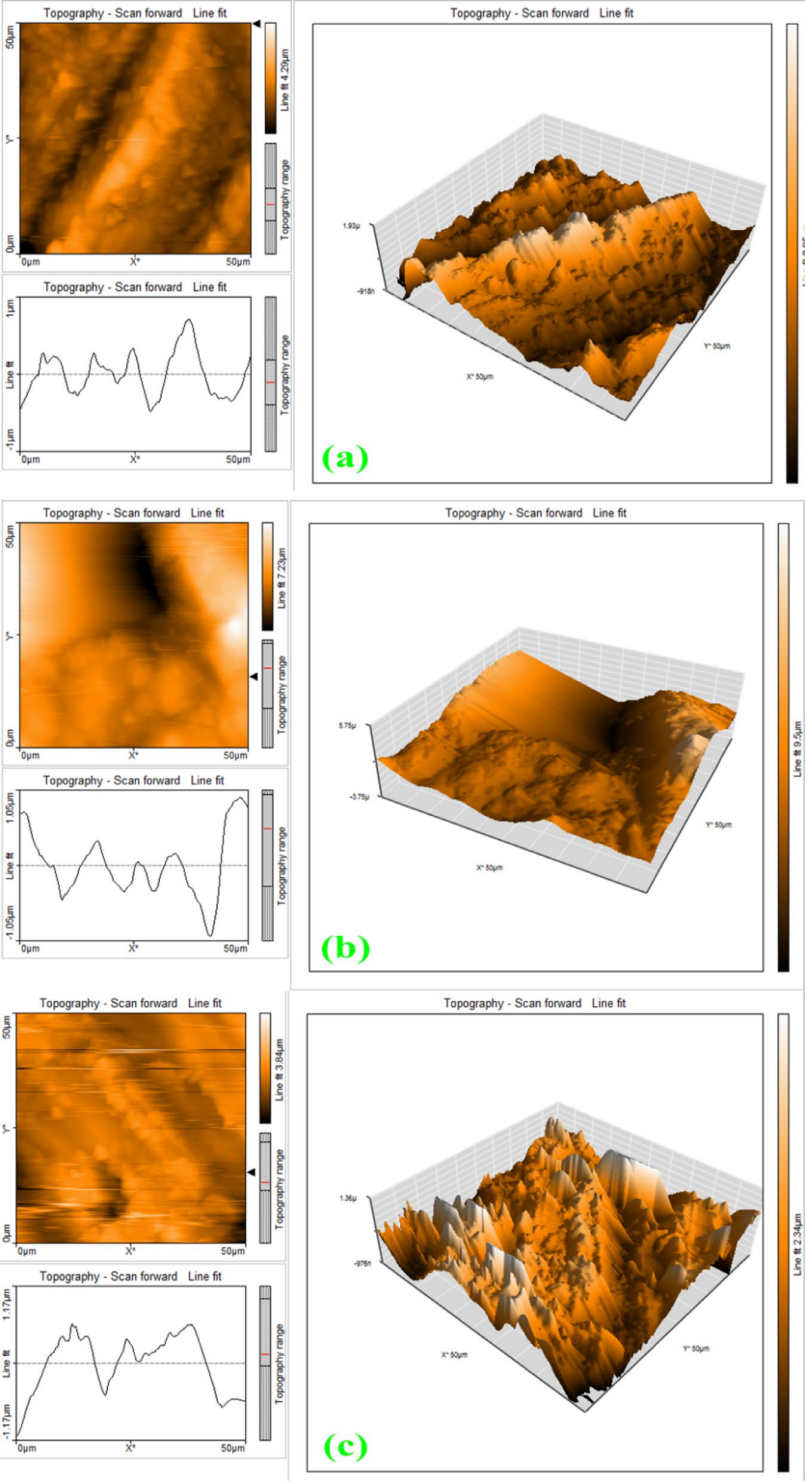
AFM images of (**a**) Polished Carbon Steel (**b**) CS immersed in the 1 M H_2_SO_4_ solution (**c**) Carbon steel + 1 M H_2_SO_4_ + 200 ppm of 0.2 M t-ZrO_2_ NPs.

Figure 9a–c shows the two-dimensional AFM images of the film-protected metal (1 M H_2_SO_4_ + inhibitor), the corroded surface (immersed in 1 M H_2_SO_4_), and the polished metal surface. In Fig. 9a–c, the equivalent three-dimensional images are also displayed. Table 1 lists the AFM parameters for refined carbon steel, along with carbon steel submerged in 1 M H_2_SO_4_, and in the inhibitor system^40^. To ensure clarity in interpreting the AFM data, the physical meanings of the symbols in Table 1 are defined as follows. The arithmetic mean roughness (S_a_) represents the average absolute deviation of surface heights from the mean plane, providing an overall measure of surface texture. The root mean square roughness (S_q_) quantifies the standard deviation of height variations and is more sensitive to extreme peaks and valleys than S_a_. The maximum peak height (S_p_) corresponds to the height of the highest asperity from the mean plane, while the maximum peak-to-valley height (S_y_) denotes the total vertical distance between the highest peak and the deepest valley within the scanned area. Collectively, these parameters describe the surface topography and enable a quantitative assessment of the inhibitor’s ability to smooth and protect the carbon steel surface.

**Table 1:** AFM data for carbon steel immersed in the inhibitor solution.

Samples	Values in (nm)			
	Maximum Peak Height (S_p_)	Root Mean Square Roughness (S_q_)	Average Roughness (S_a_)	Maximum peak-to-valley height (S_y_)
Carbon Steel Surface	3109.30	503.60	370.57	1653.30
1 M H_2_SO_4_ + Carbon Steel	2648.30	370.13	285.72	257.70
1 M H_2_SO_4_ + Carbon Steel + Inhibitor (200 ppm)	2472.30	340.42	265.73	228.50

In the corrosive media (blank), carbon steel’s average surface roughness is found to be noticeably high. However, 200 ppm of 0.2 M t-ZrO_2_ NPs inhibitor reduces this value. While it remains higher compared to the carbon steel surface which is polished, it is lower than the roughness in the corrosive medium (blank). This reduction is attributed to forming a protective layer on the carbon steel surface in the presence of the inhibitor, which appears smooth. The same trend is observed for the other three parameters such as root mean square roughness, maximum peak-to-valley height, and maximum peak height^41^.

### Electrochemical analysis

#### Weight loss method

The carbon steel’s corrosion rates (CR) when submerged in a 1 M H_2_SO_4_ solution, as well as the inhibition efficiencies (IE) with and without the presence of the 200 ppm 0.2 M t-ZrO_2_ NPs inhibitor, obtained via the weight loss method, are given in Table 2. It is noted that the 200 ppm 0.2 M t-ZrO_2_ NPs provide an inhibition efficiency of 95.2%. The inhibitory efficiency also improves with an increase in the 0.2 M t-ZrO_2_ NPs concentration^42^. This enhancement is attributed to increased surface coverage at higher inhibitor concentrations, which slows the dissolution of carbon steel. The electron-donating characteristics of the oxygen atoms in conjunction with the delocalized π-electrons, are responsible for the increased inhibitory efficiencies. This observation aligns well with findings reported by various researchers^43^.

**Table 2:** Inhibition efficiency (IE %) obtained by weight loss method.

0.2 M ZrO_2_ NPs (ppm)	CR (mpy)	IE (%)
0	5.478	-
40	2.182	62.3
80	2.105	76.2
120	2.032	81.5
160	2.001	90.7
200	1.934	95.2

#### Potentiodynamic polarization analysis

**Fig. 10:**
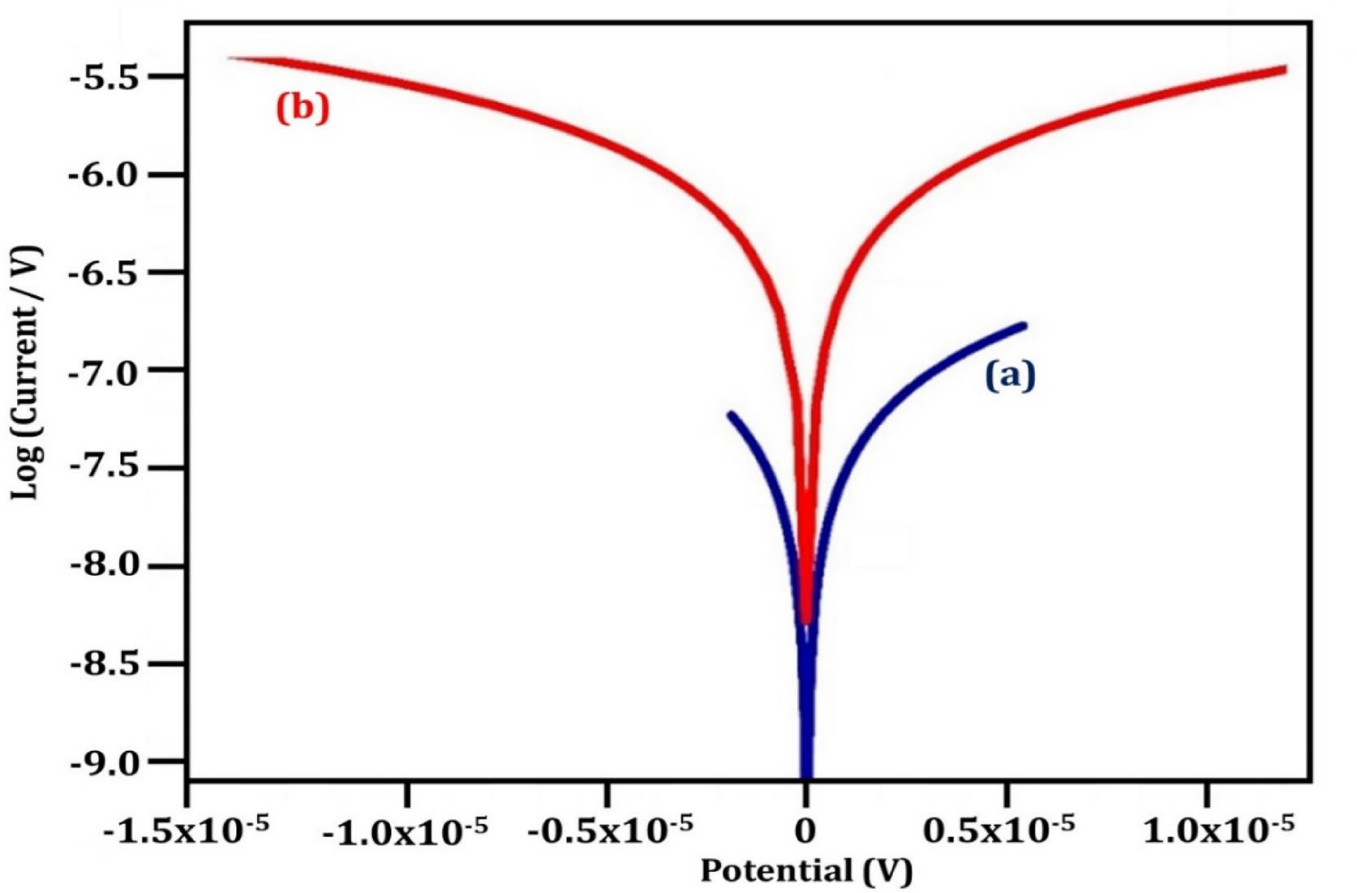
PDP Curves corrosion of CS (**a**) 1 M H_2_SO_4_ (blank) (**b**) 200 ppm of 0.2 M t-ZrO_2_ NPs.

The Tafel plots illustrating the inhibition of corrosion upon the addition of 200 ppm of 0.2 M t-ZrO_2_ NPs are presented in Fig. 10. Critical parameters such as corrosion current density (Icorr), corrosion potential (Ecorr), cathodic (b_c_) and anodic (b_a_) Tafel slopes, and linear polarization resistance (Rp) are summarized in Table 3. Adding NPs to the acidic solution decreased I_corr_ values for the system containing 200 ppm of 0.2 M t-ZrO_2_ NPs (1.987 × 10⁻⁶ µA/cm^2^) compared to the solutions without the inhibitor (0.224 × 10⁻^6^ µA/cm^2^). This decrease in corrosion When the inhibitor is present, the current density shows that the inhibitors are adsorbed onto the metal surface, preventing corrosion^44^. The measured corrosion potential value for the blank system was − 0.241 (mV vs. SCE) which increased to 0.015 (mV vs. SCE) with the inhibitor. Additionally, the linear polarization resistance (Rp) was lower for the blank system at 14,240 Ω cm^2^, compared to 24,690 Ω cm^2^ for the system with 200 ppm of 0.2 M t-ZrO_2_ NPs, suggesting the development of a shielding layer on the metal surface^40^.

**Table 3:** Potentiodynamic polarization analysis.

Inhibitor (%)	I_corr_ (µA/cm^2^)	E_corr_ (mV vs. SCE)	b_a_ (mV/dec)	b_c_ (mV/dec)	LPR (Ω·cm^2^)	IE (%)
MS + H_2_SO_4_	1.987 × 10^–6^	− 0.241	0.067	0.106	14,240	–
MS + H_2_SO_4_ + 0.2 M ZrO_2_ NPs	0.224 × 10^–6^	0.015	0.13	0.134	24,690	88.7

#### Electrochemical impedance spectroscopy analysis

**Fig. 11:**
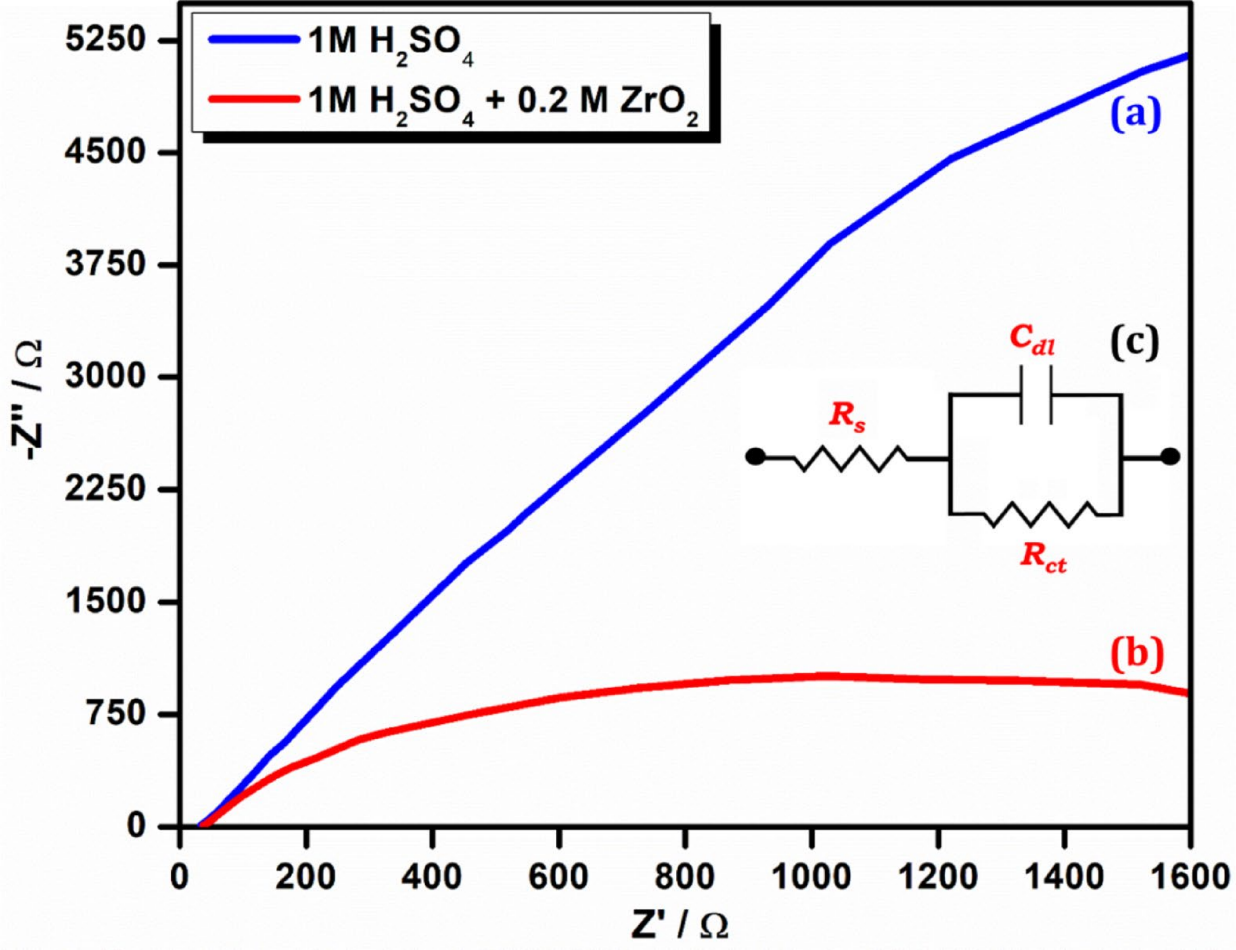
Nyquist plot for the corrosion of carbon steel (**a**) 1 M H_2_SO_4_ (blank) (**b**) 200 ppm of 0.2 M t-ZrO_2_ NPs (**c**) Electrical circuit equivalent (solution resistance: R_s_; charge transfer: R_ct_).

The development of a protective layer on the surface of carbon steel was verified by AC impedance spectra. The reduction in double-layer capacitance (C_dl_) observed in EIS studies upon the addition of t-ZrO_2_ NPs is attributed to the formation of a compact, insulating nanoparticle layer on the carbon steel surface. This layer reduces the effective area of the double layer at the metal/electrolyte interface, thereby decreasing the capacitance. The high surface area and chemical stability of t-ZrO_2_ NPs promote uniform adsorption, minimizing electrolyte access to the metal surface and enhancing the corrosion protection mechanism^41^. Figure 11a–c (Nyquist plots) displays the AC impedance spectra of submerged carbon steel in an aqueous solution containing 1 M H_2_SO_4_ both with and without the 200 ppm 0.2 M t-ZrO_2_ NPs inhibitor with electrical circuit equivalent. Table 4 displays the AC impedance parameters that were obtained from the Nyquist plots, including the double-layer capacitance (C_dl_) and charge transfer resistance (R_ct_). The charge transfer resistance (R_ct_) rose from 1,769 Ω cm^2^ to 14,715 Ω cm^2^ with the addition of 200 ppm of 0.2 M t-ZrO_2_ NPs, whereas the C_dl_ value dropped from 2.090 × 10⁷ F/cm^2^ to 0.631 × 10⁷ F/cm^2^. From 713.60 to 17,729.02, the impedance value [log (z/Ω)] increased. Table 5 shows the key findings of different coating materials previously reported and our current material under high temperature, humidity and salt water. The conclusion that a protective coating forms on the carbon steel surface is supported by these data^39,45,46^.

**Table 4:** Electrochemical impedance parameters.

System	Nyquist plot			IE (%)
	R_ct_ (Ω cm^2^)	C_dl_ (F/cm2)	Impedance log (Z/Ω)	
MS + H_2_SO_4_	1769	2.090 × 10^−8^	713.6	–
MS + H_2_SO_4_ + 0.2 M ZrO_2_ NPs	14,715	0.631 × 10^−8^	17,729.02	88.2

**Table 5:** Comparison of previously reported coating materials with the current material.

References	Coating material	External factors studied	Effect	Key findings
^4^	Sol–gel derived ZrO_2_ NPs for thermal barrier coatings	High Temperature	Phase transformation (tetragonal → monoclinic)	Reduced thermal stability and increased surface roughness
^10^	Synthesis and characterization of ZrO_2_ NPs via a simple solution route	Temperature, Humidity, Saltwater	Decreased under harsh conditions	Silver oxidation and magnetite degradation impact performance
^13^	Natural product-based inhibitors	Temperature, Humidity, Saltwater	Significant reduction in corrosion	Comprehensive overview of green inhibitors showing sustainable corrosion protection
^15^	ZrO_2_ NPs by a novel solvothermal method	Temperature Effect	Controlled phase transformation at 150–200 °C	Enhanced degradation efficiency under visible light
^21^	Green eco-friendly inhibitor for carbon steel	HCl solution, Temperature	Strong inhibition of carbon steel corrosion	Macro/micro-scale experimental and computational studies demonstrated high inhibition efficiency
^24^	anti-corrosive efficiency of NiO NPs	Temperature, Humidity, Saltwater	Significant inhibition of microbial growth	Effective in reducing corrosion rate in saltwater
^27^	Green synthesized ZrO_2_ NPs using plant extracts	Acidic medium (HCl, H_2_SO_4_), Temperature	Improved corrosion inhibition	Plant-extract-based green synthesis offers eco-friendly corrosion protection in acidic environments
^31^	Zirconium-based nanostructures	Corrosion, Biocompatibility	High chemical stability	Advanced ZrO_2_ nanostructures provide multifunctional protection including corrosion resistance in harsh environments
^17^	ZrO_2_ nanostructures for catalysis and sensing	Temperature, Humidity, Saltwater	Enhanced stability under harsh conditions	Demonstrated stability and catalytic activity in challenging environments
Current Work	t-ZrO_2_ NPs	Temperature, Humidity, Saltwater	Decreased with extreme conditions	Effective under controlled conditions but degrades in prolonged saltwater exposure

#### Cost–benefit and environmental compatibility

The green synthesis of t-ZrO_2_ NPs using *Acacia nilotica* bark extract is both cost-effective and environmentally friendly compared to traditional chemical synthesis methods. The use of plant extracts eliminates the need for toxic reducing agents and solvents, lowering production cost and toxicity. The process operates at relatively low temperatures and is scalable for industrial applications. The high inhibition efficiency (95.2% at 200 ppm) demonstrates excellent performance, justifying the cost of nanoparticle synthesis. Environmentally, the use of biomass byproducts promotes sustainability, and t-ZrO_2_ is chemically inert and non-toxic, further enhancing ecological compatibility. These features contribute to industrial relevance, especially in sectors requiring durable, eco-friendly corrosion inhibitors.

## Conclusion

0.2 M ZrO_2_ NPs were successfully synthesized using a green synthesis method with *Acacia nilotica* extract as the reducing agent, as confirmed by XRD studies. The XRD analysis showed that the 0.2 M ZrO_2_ NPs possess a tetragonal structure. FTIR analysis identified the existence of all relevant functional groups. Scanning electron microscopy (SEM) analysis showed a rock stone-like structure for the 0.2 M ZrO_2_ NPs. Furthermore, t-ZrO_2_, after being coated in carbon steel, shows a much smoother surface with reduced corrosion. EDAX confirms the existence of zirconium (Zr) and oxygen (O) in the synthesized ZrO_2_ NPs.The 0.2 M ZrO_2_ NPs exhibited significant effectiveness in inhibiting the carbon steel corrosion in an aqueous 1 M H_2_SO_4_ solution. Polarization studies revealed that these nanoparticles primarily function as anodic inhibitors, mainly influencing the anodic reaction. Weight loss experiments demonstrated that a formulation containing 200 ppm of 0.2 M ZrO_2_ NPs achieved an impressive 95.2% inhibition efficiency in mitigating the corrosion of carbon steel in the same acidic environment. Electrochemical impedance spectroscopy (EIS) measurements indicated a rise in the resistance to charge transfer (R_ct_) and a reduction in corrosion current (I_corr_) and double-layer capacitance (C_dl_), which is due to enhanced thickness of the adsorbed layer. SEM and AFM analysis revealed a smoother surface on the inhibited carbon steel in contrast to the uncontrolled samples.

## Data Availability

The datasets during and/or analyzed during the current study are available from the corresponding author upon reasonable request.
